# Supplementary material for: Does it hold weight? The perceived effects of contraceptive use on weight status in females: A mixed-methods study
Source: PLoS One. 2025 Dec 29;20(12):e0339323. doi: 10.1371/journal.pone.0339323 (PMC12747328; doi:10.1371/journal.pone.0339323)
Supplement: S1 File — (DOCX) [file pone.0339323.s001.docx]

**S3 Supplementary Material 1.**  **Questionnaire completed by respondents**

**Does it hold Weight? The perceived effects of contraceptive use on weight status in females.**

**Short Title – Perceptions of contraceptive use and weight status**

Q1**.** Age:

Q2. Height (cm):

3. Weight (kg):

4. Location:

5. Are you Premenopausal? Yes, No

6. How many hours of exercise do you complete each week?

1-2 hours

3-4 hours

5-6hours

More than 6 hours

7. Are you currently taking oral hormonal contraceptives?

8. Have you ever taken hormonal contraceptives in your lifetime?

9.How long have you used contraceptives (this includes ALL methods not just your current one if others apply):

Less than 6 months

6 months -less than 2years

2-5years

More than 5 years

10. Have you ever had any breaks between methods and if so how long were these breaks?

No

Less than 1 month

Less than 3 months

Less than 6 months

Less than 1 year

Less than 2 years

More than 2 years

11. What was the main reason for you taking oral hormonal contraceptives?

12. Are there any other reasons you take oral contraceptives (Please tick all that are appropriate)

Prevent pregnancy

To prevent a period

Inconsistent cycles

To prevent period pain

Other.

12a. If you selected other, please specify:

13. Which type of hormonal contraception do you currently use?

Pill – continuous/extended

Pill – progesterone only

Pill – combined (oestrogen-progesterone)

Pill unsure which one

Contraceptive implant

Other or not listed

IUS intrauterine system or hormonal coil

IUD Intrauterine device or coil (copper)

Contraceptive patch

14. How long have you been on/taking your most recent method of oral hormonal contraceptive?

Less than 6 months

6 months – less than 2years

2-5years

More than 5 years

15. Do or did you experience any side effects from your current contraceptive method?

Yes

No

Side effects

Bleeding irregularities

Weight gain

Mood changes

Breast tenderness

Headaches

Nausea

Other

16. Did you experience any changes to your body immediately after taking any form of oral hormonal contraceptive?

Yes

No

17. If yes, please specify (which methods and the outcome=weight loss, weight gain etc; for example vaginal ring led to weight loss)

18. If yes, please specify (which methods and the outcome=weight loss, weight gain etc; for example vaginal ring led to weight loss)
