# Supplementary material for: Does it hold weight? The perceived effects of contraceptive use on weight status in females: A mixed-methods study
Source: PLoS One. 2025 Dec 29;20(12):e0339323. doi: 10.1371/journal.pone.0339323 (PMC12747328; doi:10.1371/journal.pone.0339323)
Supplement: S2 File — (DOCX) [file pone.0339323.s002.docx]

**S4: Supplementary Material 2: Timeline online interview questions**

**Does it hold Weight? The perceived effects of contraceptive use on weight status in females.**

**Short Title – Perceptions of contraceptive use and weight status**

Interview Overview

Introduction

Thank you for volunteering to take part in our study. We are interested in understanding the impact on contraception on your perceptions of weight loss/gain and more wider general life factors that influence or was influenced by taking contraception. Therefore, we will be doing a bit of a life history interview where I will ask you to think back to when you first started to think about starting to take contraception and then we will work chronologically to now. The interview should last approximately 30-45 mins at the most. If at any point you’re not 100% sure of the question I am asking or if you need more time to answer the question, please don’t hesitate to ask for clarification or ask for more time to think about your response. If at any point you don’t want to answer my questions just say and we will move on. If at any point you wish to stop the interview, please just say and we will do so immediately.

Do you have any questions?

Are you happy for me to start?

Sporting history if any….

Tell me about your engagement with sport and physical activity (aiming to draw out here what sport and what standard they competed at)

How many hours per week

What intensity

**Question 1)** Please can you think right back to when you first started to think about taking contraception. How old were you and what was happening in your life at the time?

Ask – motives for taking contraception.

If not covered when answering this question, then ask the following:

- 1. Where they influenced by peers/school/parents to take oral hormonal contraceptives
  2. Any other medications they were on
  3. Current physical activity status
  4. Nutrition/diet - what was your nutrition like?
  5. Were you happy with your body image at the time?
  6. Were you trying to lose weight at this time?
  7. Any other life events occurring?

**Question 2)** Can you now talk me through key stages of your life where you were still taking contraception. E.g. University, Job, marriage, kids etc etc. (go through the below questions for each life stage).

- 1. What was influencing you to take the contraception at this time?
  2. Any other medications they were on
  3. Current physical activity status
  4. Nutrition/diet - what was your nutrition like?
  5. Were you happy with your body image at the time?
  6. Were you trying to lose weight at this time?
  7. Any other life events occurring?

Ask the above questions again for each key stage of their life history, ensuring that the timeline of life is captured, repeating until present day is reached.

**Question 3)** Do you think overall, the use of contraception impacted on your weight? If yes, specifically when?

- 1. How easy do you feel it is to lose weight over the course of taking oral hormonal contraception
  2. Do you think there was a specific contraceptive method you found it more difficult/easier to loose weight on

If they have never attempted to loose weight- ask them what their current belief/ insights are?

**Question 4)** is there anything you think I have missed?

Thank you for speaking with me today.- Ask them politely to share the link to the questionnaire with others.

**Detail for timeline questions to gain a full understanding of the participants reasons for contraceptive use :**

Overview of full contraception history (aim here to get a full history of contraceptive intake over their lifetime

- 1. How old were they when they first started taking oral hormonal contraceptives?
  2. Reasons for taking it
  3. Where they influenced by peers/school/parents to take oral hormonal contraceptives
  4. Any other medications they were on

1. Any important life changes which occurred at a similar time as starting the contraception- It is probably good to go over a timeline of their life and pinpoint any significant life changes which have happened
   1. New job
   2. New relationship
   3. COVID
   4. Kids
   5. Loosing job
   6. Bereavement
2. Nutrition/diet knowledge
   1. What do they do for work? (Is it health orientated?)
   2. Try and get insight into their overall diet now
   3. Then try and get them to think back and ask if their diet was similar then
3. What is their current physical activity status?
   1. Have they always had this level of activity?
   2. Changed sports?
4. Weight loss attempts
   1. View on any weight changes at the onset of taking oral hormonal contraceptives
   2. Have they ever attempted to lose weight on vs off contraceptive
   3. What kind of diet did they go on if any?
   4. Type of diet
   5. Did they manage loose weight?
   6. How much did they loose?
   7. Did they maintain this weight loss?
   8. Do you feel those experiences were different? On vs off contraceptives
   9. How easy do you feel it is to lose weight over the course of taking oral hormonal contraception
