# Supplementary material for: Does it hold weight? The perceived effects of contraceptive use on weight status in females: A mixed-methods study
Source: PLoS One. 2025 Dec 29;20(12):e0339323. doi: 10.1371/journal.pone.0339323 (PMC12747328; doi:10.1371/journal.pone.0339323)
Supplement: Attachment — Submitted filename: Response to reviewers FINAL.docx [file pone.0339323.s003.docx]

| **Reviewers Comments** |  |
| --- | --- |
| **Your Comment / Recommendation** | **Revision** |
| 1.Please ensure that your manuscript meets PLOS ONE's style requirements, including those for file naming. The PLOS ONE style templates can be found at  https://journals.plos.org/plosone/s/file?id=wjVg/PLOSOne_formatting_sample_main_body.pdf and  <https://journals.plos.org/plosone/s/file?id=ba62/PLOSOne_formatting_sample_title_authors_affiliations.pdf> | All of the formatting for this manuscript has now been aligned to PLOS ONE’s style this includes:   - Cover Page - Referencing (numerical) - Fig labels in-text - Fig labels diagram - Table Formatting |
| 2. Please ensure that you refer to Figure 1 and 3 in your text as, if accepted, production will need this reference to link the reader to the figure. | Figures have been added to the text |
| We note that there is identifying data in the Supporting Information file “Data Submission.xlsx”. Due to the inclusion of these potentially identifying data, we have removed this file from your file inventory. Prior to sharing human research participant data, authors should consult with an ethics committee to ensure data are shared in accordance with participant consent and all applicable local laws.  Data sharing should never compromise participant privacy. It is therefore not appropriate to publicly share personally identifiable data on human research participants. The following are examples of data that should not be shared:  -Name, initials, physical address  -Ages more specific than whole numbers  -Internet protocol (IP) address  -Specific dates (birth dates, death dates, examination dates, etc.)  -Contact information such as phone number or email address  -Location data  -ID numbers that seem specific (long numbers, include initials, titled “Hospital ID”) rather than random (small numbers in numerical order)  Data that are not directly identifying may also be inappropriate to share, as in combination they can become identifying. For example, data collected from a small group of participants, vulnerable populations, or private groups should not be shared if they involve indirect identifiers (such as sex, ethnicity, location, etc.) that may risk the identification of study participants.  Additional guidance on preparing raw data for publication can be found in our Data Policy (https://journals.plos.org/plosone/s/data-availability#loc-human-research-participant-data-and-other-sensitive-data) and in the following article: http://www.bmj.com/content/340/bmj.c181.long.  Please remove or anonymize all personal information (<specific identifying information in file to be removed>), ensure that the data shared are in accordance with participant consent, and re-upload a fully anonymized data set. Please note that spreadsheet columns with personal information must be removed and not hidden as all hidden columns will appear in the published file.  4. If the reviewer comments include a recommendation to cite specific previously published works, please review and evaluate these publications to determine whether they are relevant and should be cited. There is no requirement to cite these works unless the editor has indicated otherwise.   Additional Editor Comments: **You are requested to read the reviewers comments carefully and submit the point wise reply against each raised query. For comments you do not agree and feel that the original submission is justified reference to your study, must also be replied in detail.** | All data which contains identifiable information has now been redacted. |
| 1. Mixed-Methods Integration Gap: o While the authors adopt a sequential explanatory design (quantitative followed by qualitative), the integration of findings across methods is largely descriptive and lacks depth. There is limited evidence of how the qualitative interviews build upon or help explain specific patterns observed in the survey data. o Recommendation: Clearly articulate in the results and discussion sections how the qualitative themes help to confirm, challenge, or explain the quantitative findings. Consider using a joint display or matrix to demonstrate this integration explicitly. | We thank the reviewer for this constructive observation. In line with your recommendation, we have strengthened the integration of the quantitative and qualitative strands in both the Results and Discussion. Specifically:   1. **Results section** – We now present survey findings alongside corresponding interview insights, and have added a joint display table (Table 3) that aligns survey responses with emergent qualitative themes and provides an integrated interpretation. This makes explicit how interview data confirm, expand upon, or challenge survey patterns (e.g., survey reports of weight management difficulties clarified as lifestyle-related in interviews, with DMPA consistently identified as an exception). 2. **Discussion section** – We revised the opening paragraphs to interpret these integrated findings, highlighting how mixed-methods analysis refined the survey results. While 42% initially reported greater difficulty with weight management on contraceptives, qualitative reflection suggested many of these difficulties were attributable to lifestyle and psychosocial factors. However, interviews consistently highlighted DMPA as a direct driver of perceived weight struggles. This integration demonstrates how qualitative data added depth, nuance, and explanatory value beyond descriptive survey results.   We believe these revisions substantially enhance the coherence and depth of our mixed-methods design and directly address the reviewer’s request for clearer articulation of how qualitative themes build upon and explain quantitative findings. |
| 2. Sampling and Representativeness: o The study claims to generalize to "females of reproductive age," but the sample is predominantly from the UK and appears to over-represent physically active women (e.g., 44.8% report >6 hours of exercise/week). This could bias perceptions of weight management and may not reflect the general population. o Recommendation: Discuss the sampling bias and its implications more clearly in the limitations section. Consider tempering generalizations and clarifying that findings may be most relevant to physically active, health-conscious populations. | We agree with the reviewer’s observation and have clarified the representativeness of our sample.  **Limitations section** - We now explicitly acknowledge that participants were predominantly UK-based and highly active, with nearly half reporting more than six hours of exercise per week. We note that this likely reflects a health-conscious population and that findings should not be assumed to generalise to all females of reproductive age. The following paragraph has been added: *“A further limitation is the representativeness of our sample. Participants were predominantly based in the UK and highly active, with 44.8% reporting more than 6 hours of exercise per week. This profile likely reflects a health-conscious population who may be more aware of or sensitive to weight changes than the general population. As such, findings should not be generalised to all females of reproductive age, but may be most applicable to physically active or health-oriented groups. Future research should recruit more diverse samples to explore whether perceptions differ across less active populations and across different cultural contexts”.*   - **Abstract and Conclusion** - We tempered our language to reflect this limitation. In the Abstract, we now state that the sample was predominantly UK-based and physically active. In the Conclusion, we specify that findings may be most relevant to active or health-conscious populations and highlight the need for replication in more diverse samples. The text has been amended as following: “*fifteen predominantly UK-based females”*   These revisions ensure that the potential sampling bias is clearly acknowledged and that our conclusions are appropriately contextualised. |
| 3. Operationalization of “Weight Management Struggles”: o The manuscript heavily relies on participants’ self-reported struggles with weight loss or gain without defining how "struggle" was measured or whether this referred to actual weight change or subjective difficulty. o Recommendation: Provide clarity in the methods on how “struggle” was conceptualized and whether validated scales or anchor questions were used. Otherwise, the construct remains vague and open to interpretation. | We thank the reviewer for this helpful comment. We have now clarified how “weight management struggles” were conceptualised in the study. Specifically:   - **Methods section**– We added the following sentence: *“In this study, ‘weight management struggles’ were defined as participants’ self-reported experiences of attempting to lose weight but perceiving no change on the scales, or perceiving greater difficulty in managing weight whilst on contraceptives. This construct was subjective and designed to reflect perceptions, in line with the study’s aim to explore female experiences, rather than objectively measured weight change.”* - **Limitations section**– We added: *“Finally, our operationalisation of ‘weight management struggles’ was based on subjective perceptions rather than objectively measured weight change, and should therefore be interpreted as reflective of perceptions rather than physiological outcomes.”*   These revisions make explicit that our measure was subjective, consistent with the study’s aim to capture perceptions, while also acknowledging this as a limitation. |
| 4. Interpretation of DMPA-Related Weight Gain: o While DMPA is repeatedly identified as associated with weight gain, the discussion lacks nuance regarding dose, duration, and metabolic factors. The attribution is largely anecdotal and may reflect confirmation bias. o Recommendation: Strengthen this section by discussing the limitations of self-reported weight change, the potential for recall bias, and contrasting findings from prospective studies on DMPA. Consider the role of coexisting lifestyle changes. | We thank the reviewer for highlighting this valuable point. We have now expanded the Discussion section to provide greater nuance and contextualisation regarding DMPA-related weight gain, drawing on prospective evidence and acknowledging limitations of self-report. Specifically, we added the following paragraph immediately after the discussion of DMPA-related mechanisms:  *“It is important to interpret these DMPA-related findings in light of methodological and contextual limitations. The current data are based on retrospective self-report, and therefore may be influenced by recall bias, particularly regarding the timing and extent of weight changes. Perceived weight gain may not always align precisely with objective measurements, and prior prospective studies provide mixed evidence. For example, Berenson and Rahman (2009) reported that over a 36-month period, DMPA users experienced significantly greater increases in body weight (+5.1 kg), fat mass (+4.1 kg), and central-to-peripheral fat ratio compared with oral and non-hormonal contraceptive users, suggesting a potential biological influence of DMPA on adiposity. In contrast, Bonny et al. (2011) examined adolescents initiating DMPA and found that early weight gain (>5% within six months) predicted continued excessive weight gain at 12–18 months. While this provides valuable insight into early predictors of weight gain, it should be noted that this study was conducted in adolescents, whose hormonal and behavioural profiles differ from adult women in the present study. Such discrepancies between studies likely reflect differences in participant age, duration of use, and individual metabolic or lifestyle factors. Overall, the current findings should therefore be viewed as reflecting a multifactorial process in which both pharmacological effects and coexisting lifestyle behaviours contribute to perceived weight change.”*  This addition explicitly addresses the reviewer’s request by:   - contrasting self-reported data with prospective studies, - discussing recall bias and methodological constraints, and - noting that one of the cited studies focused on adolescents, distinguishing it from our adult sample.   We believe this provides the nuance and balance the reviewer requested while situating our findings within the broader evidence base. |
| 5. Use of Social Ecological Model (SEM): o Although SEM is introduced as the framework, its application in the findings is inconsistent. The mapping of themes onto SEM levels (e.g., interpersonal, societal) seems forced at times and lacks theoretical depth. o Recommendation: Ensure that SEM is systematically applied across all themes with clearer justification. Avoid vague attributions (e.g., "societal level" for stress) and explain how each level influenced contraceptive perception and behavior. | We appreciate this thoughtful feedback. To strengthen theoretical consistency and ensure the Social Ecological Model (SEM) is systematically applied, we have revised both the Methods and Findings and Discussion sections.  In the **Methods**, we now clarify how the SEM guided both coding and interpretation:  “The Social Ecological Model (SEM) was used deductively to guide coding and theme organisation, while allowing inductive sub-themes to emerge within each level. Individual-level influences captured personal beliefs, physiological experiences, and self-directed behaviours. Interpersonal factors referred to relationships and immediate social contexts (e.g., partners, peers, coaches) shaping contraceptive experiences and weight perceptions. Community and societal levels encompassed institutional or cultural influences such as workplace demands, university environments, and sociocultural body ideals. This framework allowed multi-layered interpretation of how biological, behavioural, and contextual factors interact to influence perceived weight change.”  In the **Findings and Discussion**, we added an integrative paragraph at the start of the section to demonstrate how the SEM was applied consistently across the analysis:  “Findings are interpreted through the Social Ecological Model to highlight how influences on perceived weight management operate across multiple, interacting layers. At the individual level, participants described physiological sensations, appetite changes, and emotional responses related to contraception. The interpersonal level encompassed support networks and social comparison, such as conversations with partners, peers, or trainers that shaped interpretations of weight gain. The community level included transitions into new environments—university, employment, or sporting settings—that altered lifestyle routines. Finally, societal influences such as cultural ideals of thinness and public discourse around contraception framed participants’ expectations and self-evaluation. Applying the SEM systematically in this way illustrates that perceptions of contraceptive-related weight change are co-constructed through personal, relational, and structural contexts rather than arising from physiology alone.”  These revisions ensure consistent and theoretically grounded application of the SEM throughout the manuscript. |
| Minor Comments |  |
| 1. Background in Introduction: o The introduction would benefit from a stronger contextual background on the significance of perceived weight changes in contraceptive users, especially linking this perception to contraceptive adherence and health outcomes. A more focused rationale would help set up the need for this mixed-methods study. | We thank the reviewer for this valuable suggestion. We have now strengthened the Introduction to provide a clearer rationale for the study and to explicitly link perceptions of weight change with contraceptive adherence, health outcomes, and the need for a mixed-methods approach. Specifically, we added the following paragraph after the discussion of psychological and behavioural barriers to oral contraceptive use (Westhoff et al., 2007; Littlejohn, 2013):  *“Perceived weight change, even in the absence of objectively measured gain, has significant implications for contraceptive adherence and broader health outcomes. Studies have shown that concerns about weight gain are among the most frequently cited reasons for discontinuation or avoidance of hormonal contraceptives (Westhoff et al., 2007; Littlejohn, 2013). Discontinuation due to perceived side effects can increase the risk of unintended pregnancy, compromise reproductive autonomy, and contribute to contraceptive method switching, which may further disrupt menstrual regularity and hormonal stability (Smith & Harris, 2023). Moreover, perceptions of reduced control over weight can negatively impact body image, self-esteem, and engagement with health-promoting behaviours such as physical activity and balanced nutrition. Understanding these perceptions—and whether they are grounded in physiological changes or influenced by lifestyle and psychosocial factors—is therefore essential for improving contraceptive counselling, adherence, and women’s overall well-being. This underscores the need for a mixed-methods approach to capture both the prevalence and lived experience of perceived weight change among contraceptive users.”*  This addition provides a stronger theoretical and applied context for the study, clearly articulating the significance of perceived weight change for contraceptive use and women’s health. It also reinforces the justification for adopting a mixed-methods design to address both the prevalence and lived experiences of these perceptions. |
| 2. Study Location Missing in Abstract: o The abstract should clearly state where the study was conducted (e.g., United Kingdom). This adds context and helps readers assess the generalizability of findings. | This has been amended to state the population is predominantly UK based. |
| 3. Repeated References: o Some references appear more than once with minimal distinction (e.g., Brown & Clegg 2010a and 2010b; Gallo et al. 2014a and 2014b). Please check for redundancy and consolidate citations where appropriate. | Thank you for this observation. We have amended redundant referencing. |
| 4. Outdated Prevalence References: o The introduction cites contraceptive prevalence using data from older sources (e.g., WHO 2014, Alkema et al. 2013). Consider updating this with more recent national or global estimates (e.g., WHO or UNFPA 2022 reports). | We thank the reviewer for this helpful observation. We have updated the introduction to include the most recent global data on contraceptive prevalence. Specifically, we now reference the **World Health Organization (2022)** *Impact of Contraceptive Use on Women’s Health and Socioeconomic Status: Evidence Brief*, which reports global modern contraceptive use among women of reproductive age. This replaces the earlier WHO (2014) reference. The revised sentence now reads:  *“Recent estimates indicate that in 2022, approximately 874 million women of reproductive age (15–49 years) were using modern contraceptive methods worldwide (World Health Organization, 2022).”*  This update ensures the manuscript reflects current global prevalence figures and aligns with the most up-to-date WHO reporting. |
| 5. Questionnaire Validation: o While pilot testing is mentioned, there is no clear description of how the questionnaire was validated. Was face validity or content validity assessed formally? Clarifying this would strengthen the reliability of the quantitative data. | We thank the reviewer for this constructive suggestion. We have now clarified in the *Methods* section how the questionnaire was validated to establish both **face** and **content validity**. Specifically, members of the research team, who have expertise in exercise physiology, women’s health, and behavioural nutrition, reviewed all questionnaire items to confirm their alignment with the study aims and to ensure clarity, coherence, and coverage of relevant domains (content validity). The questionnaire was then **pilot tested with five eligible participants** to assess comprehension, flow, and usability (face validity). Minor wording changes were made based on this feedback to improve participant understanding and overall usability. The revised paragraph now reads:  *“‘Jisc Online Surveys’ (formerly Bristol Online Surveys; Joint Information Systems Committee, Bristol, UK) was used to create the questionnaire and collect responses anonymously. The questionnaire underwent a two-stage validation process to ensure face and content validity. Initially, members of the research team (academics in exercise physiology, women’s health, and behavioural nutrition) reviewed all items to confirm alignment with the study aims and assess clarity, coherence, and coverage of relevant domains (content validity). The instrument was then pilot tested with five eligible participants to evaluate comprehension, flow, and usability (face validity). Minor wording changes were made based on this feedback to enhance clarity and participant understanding.”*  This revision provides a clear description of the validation process and strengthens the methodological transparency and reliability of the quantitative component. |
| 6. Abbreviation Use (e.g., DMPA): o Ensure that all abbreviations are written out in full at first mention (e.g., “DMPA” should be “Depot Medroxyprogesterone Acetate (DMPA)” initially). This helps readers who are not familiar with contraceptive terminology. | We thank the reviewer for this observation. We confirm that all abbreviations, including *Depot Medroxyprogesterone Acetate (DMPA)*, were already written out in full at their first mention within the manuscript. “, such as depot medroxyprogesterone acetate (DMPA)”  However, upon review, we noted that *Body Mass Index (BMI)* had not been defined at first mention and have now corrected this. All abbreviations are now introduced appropriately to ensure clarity for readers. |
| 7. Title Clarity: o The main title “Does it hold weight?” is catchy but somewhat vague. Consider adding a more descriptive subtitle to improve clarity (e.g., “Perceived Effects of Contraceptive Use on Weight in Females: A Mixed-Methods Study”). | We thank the reviewer for this comment. We appreciate the suggestion and agree that clarity is important. However, we have retained the original title, *“Does it hold weight?”*, as it has a clear link to the study focus and has been paired with a descriptive subtitle — *“The perceived effects of contraceptive use on weight status in females: A mixed-methods study.”* Together, these provide both engagement and clarity while maintaining alignment with PLOS ONE’s style and accessibility for a broad readership. |
| 8. Grammar and Formatting: o Minor grammatical issues and typographical errors are scattered throughout the manuscript (e.g., missing articles, extra spaces, inconsistent tenses). A thorough proofread is recommended. | We thank the reviewer for highlighting this. The entire manuscript has now undergone a comprehensive proofread and edit for grammar, typographical consistency, and tense alignment. Specific revisions included:   - Correction of missing or misplaced articles (e.g., “the,” “an,” “a”). - Removal of redundant spaces and line breaks. - Standardisation of tense across the manuscript, ensuring the past tense is consistently used when describing study procedures and results. - Harmonisation of terminology (e.g., “contraceptive use” and “weight management struggles” used consistently throughout). - Review of punctuation, especially around parentheses and em dashes, to comply with PLOS ONE style. We believe these revisions have resolved all grammatical and formatting inconsistencies noted by the reviewer. |
| 9. Inconsistent Terminology: o The manuscript uses terms like “weight gain,” “weight status,” and “weight management struggles” interchangeably. These should be clearly defined and used consistently to avoid confusion. | We thank the reviewer for this helpful observation. We have carefully reviewed the manuscript to ensure consistent use of terminology.  In the *Methods* section, we have clarified how each term is applied throughout the paper. Specifically, *“weight management struggles”* refers to perceived difficulty in controlling or reducing body weight while using contraceptives, *“weight status”* is used to describe general body weight outcomes, and *“weight gain”* is reserved for instances where participants explicitly reported an increase in weight. The following sentence has been added to the *Methods* section:  *“The term ‘weight management struggles’ is used specifically to describe perceived difficulty in controlling or reducing body weight while using contraceptives, whereas ‘weight status’ refers to general body weight outcomes, and ‘weight gain’ is reserved for instances where participants explicitly reported an increase in weight.”*  We have also amended some aspect of the discussion session to keep terminology aligned with the operational definitions added into the methods section.  *“The DMPA injection was consistently reported to contribute to weight management struggles and difficulty losing weight.”*  *“Whilst the results of this study highlight the potential effects of contraception—particularly DMPA—on weight management struggles and perceived difficulty in losing weight”*  *These narratives highlight the complex interplay between contraception use and life stressors such as work, stress, lifestyle changes, and major life events in influencing perceived weight management.*  *“...those who received DMPA perceived the contraceptive as contributing to weight management struggles and, in some cases, weight gain, independent of other confounding variables.”*  *“...clinicians, coaches, and recreationally active females should consider the most appropriate contraceptive type for their circumstances and also reflect on life status and current events as potential contributors to perceived weight management struggles or weight gain.”*  These revisions ensure clear and consistent terminology across the manuscript. |
| 10. Figures and Tables: • Figure references (e.g., “FIGURE 2 NEAR HERE”) appear as placeholders. Ensure figures are properly inserted, numbered, captioned, and clearly explained in the text. Table formatting could also be improved for clarity. | All figures have now been inserted, numbered, captioned and explained/referred in-text. |
| 11. Use of First-Person Language: • Phrases such as “we are aware” appear in the manuscript. Consider revising these to maintain a neutral academic tone unless first-person language is permitted by journal guidelines. | *“The results are presented in a way that aligns with the study’s analytical framework; however, readers may engage in their own naturalistic generalisability”*  *“An established six-step reflexive thematic analysis approach (Braun & Clarke, 2021) was followed. The analyst familiarised themselves with the data and generated initial codes.”*  *“Themes were subsequently refined through discussion within the research team to ensure credibility and trustworthiness (Braun & Clarke, 2006).”*  *“By integrating survey results with interview themes (Table 3), the analysis refined the quantitative patterns.”* |
| 12 Consider including basic inferential statistical analysis, such as calculating odds ratios or chi-square tests, to enhance the quantitative interpretation of survey findings. If appropriate, consult a statistician to strengthen the analytical rigor. | We have run a chi squared test to compare whether participants felt it was easier, harder or no different in weight loss. (χ ^2^ (2, N = 196) = 92.86, *p* <0.001) there was a significant effect for perceived difficulty to lose weight whilst taking oral hormonal contraceptives. No chi squared test however could be run on type of contraceptive use due to sample size (see table for individual group sample size). **Total participants who provide contraceptive alongside weight loss experience (N = 94)** \| \| \| \| \|; \| Pill Progesterone Only \| Contraceptive Implant \| IUS or hormonal coil \| Pill Combined (oestrogen and progesterone) \| Pill (unsure which one); Easier \| 1 \| 0 \| 0 \| 0 \| 0; No Diff \| 6 \| 4 \| 2 \| 37 \| 6; Harder \| 9 \| 4 \| 2 \| 21 \| 2 |
| **Reviewer 2** |  |
| Lifestyle Confounding Factors Not Assessed: The quantitative survey did not include questions about nutritional patterns (e.g., meal frequency, diet quality) or detailed physical activity classification (e.g., light/moderate/vigorous activity). These omissions are significant given the study’s focus on weight perception and management. | We thank the reviewer for this comment. The purpose of the current study was to explore **women’s perceptions and interpretations** of weight management while using contraceptives, rather than to quantify behavioural determinants of weight change. Because this was a **retrospective, perception-based design**, collecting data on diet quality or physical activity intensity would have introduced substantial recall bias; such data are unreliable even when collected in real time, and far more so when recalled over long periods.  Crucially, understanding perception is central to contraceptive adherence. Women’s beliefs about weight change—whether physiologically accurate or not—directly influence behaviours such as **method discontinuation, inconsistent use, or avoidance of hormonal contraception altogether**. Exploring perceptions therefore provides insight into real-world decision-making and adherence patterns that objective physiological data alone cannot explain. This qualitative-quantitative approach also allowed us to gather a large dataset efficiently while capturing the contextual and psychosocial factors shaping these perceptions.  We have clarified this rationale and added the following statement to the *Discussion*:  *“While the present study explored lifestyle influences qualitatively, objective assessment of diet quality or activity intensity was not feasible due to the recall bias inherent in retrospective assessment. Importantly, women’s perceptions of how contraception affects weight are central to real-world adherence, influencing discontinuation, inconsistent use, and method switching. Understanding these perceptions is therefore as important as, if not more important than, measuring physiological change. Understanding these lived experiences is therefore essential for improving contraceptive counselling and supporting sustained use. Future prospective research could complement these findings with objective behavioural measures collected in real time.”* |
| No Objective Measures of Body Composition: The study relies entirely on self-reported weight-related perceptions without data on body composition (fat mass vs. muscle mass) or clinical indicators (e.g., presence of edema or fluid retention, which are common with some contraceptives). This limits the physiological interpretability of the findings. | We appreciate the reviewer’s observation. The study was intentionally designed to capture **subjective experiences** and perceived mechanisms rather than to quantify physiological change. Women often make contraceptive decisions based on *how they feel* their body is responding, not on measured body composition. Understanding these perceptions is critical for improving adherence and communication between patients and clinicians.  This is address in the edited paragraph in the previous response |
| Missing Pharmacological Details: The manuscript does not specify which types of progestins were included in the combined oral contraceptives (COCs) used by participants. Since progestins have differing metabolic effects, this information is critical and should be reported. | We thank the reviewer for this observation. The purpose of the current study was to examine women’s **perceptions and experiences** of weight management while using contraceptives, rather than to assess pharmacological or metabolic mechanisms. As the study was retrospective and self-reported, many participants were unable to recall the specific name or formulation of their contraceptive method, which limited accurate reporting of individual progestin types.  This limitation is now acknowledged in the *Discussion*.  Importantly, the only contraceptive consistently associated with perceived weight-related effects in this cohort was **Depot Medroxyprogesterone Acetate (DMPA)**, which has a standardised formulation across users. As such, detailed pharmacological differentiation between oral contraceptive formulations would not have altered the interpretation of our findings.  *“Because this study relied on retrospective self-report, many participants could not recall the specific name or formulation of their contraceptive, which limited precise identification of progestin types However, it is important to note those who received DMPA which has a uniform composition perceived the contraceptive as contributing to weight management struggles and, in some cases, weight gain, independent of other confounding variables”* |
| Clinical Inconsistency Not Addressed: One participant reportedly used COCs to stop migraines, which contradicts current clinical guidelines that contraindicate combined hormonal contraceptives in women with migraine-especially with aura - due to elevated stroke risk. This discrepancy should be clarified or contextualized. | We thank the reviewer for this observation. However, assessing clinical adherence to prescribing guidelines was beyond the scope of this perception-based study. The data were **retrospective and self-reported**, and therefore reflect participants’ recollections and personal interpretations rather than verified medical records. The mention of one participant using combined oral contraceptives (COCs) for migraine management was included to represent their lived experience, not to comment on clinical appropriateness. |
| Anonymity of Qualitative Data: While the interview excerpts are rich and informative, some are highly personal and may allow participants to be identified. It should be clarified whether participants gave explicit consent for their quotations to be published. | We thank the reviewer for raising this point. All participants provided **written informed consent** prior to participation, which included explicit permission for anonymised quotations to be used in publications and presentations. Identifying information was removed from all transcripts, and participant quotes are presented using only coded identifiers. |
| Recommendations for Revision: -Include specific details about contraceptive formulations (especially the progestins used). -Clarify ethical procedures regarding qualitative data publication. -Add discussion about the lack of objective anthropometric or clinical data (e.g., edema). -Consider a stronger discussion of lifestyle confounders and suggest their inclusion in future research tools. -Clarify or revise the mention of COC use for migraine management to align with safety guidelines. | We thank the reviewer for their thorough evaluation and constructive feedback. We have carefully reviewed and addressed each of the points raised throughout our detailed responses above. Relevant clarifications and revisions have been incorporated into the *Methods*, *Discussion*, and *Limitations* sections. We appreciate the reviewer’s thoughtful insights, which have helped to strengthen the overall clarity and rigour of the manuscript. |
